# Supplementary figures and images for: High-Dimensional Single-Cell Analysis Reveals Coordinated Age-Dependent Neuroinflammatory Microglia-T cell Circuits in the Brain
Source: bioRxiv. 2025 Dec 13:2025.12.10.693494. Preprint. [Version 1] doi: 10.64898/2025.12.10.693494 (PMC12712942; doi:10.64898/2025.12.10.693494)

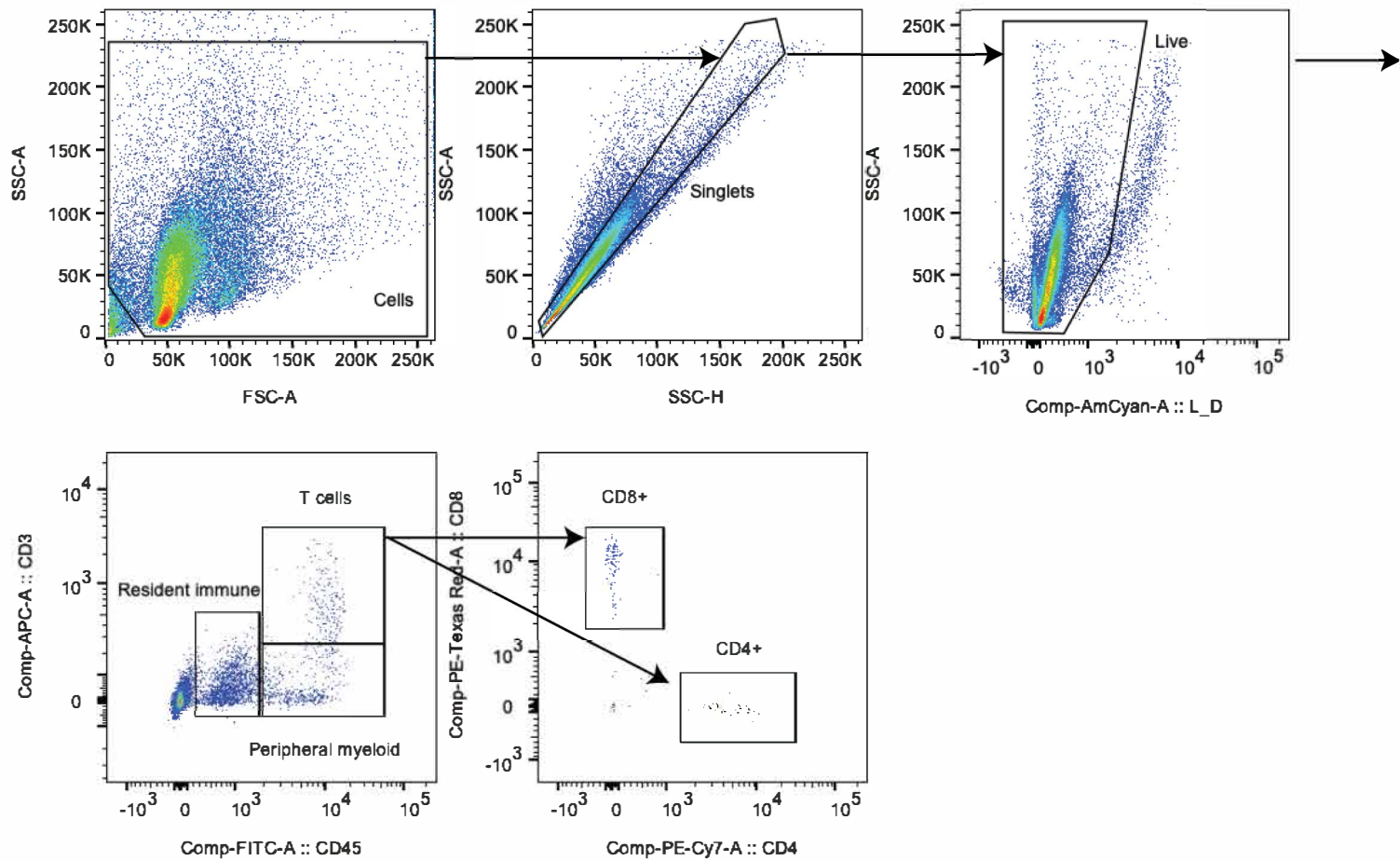

**A**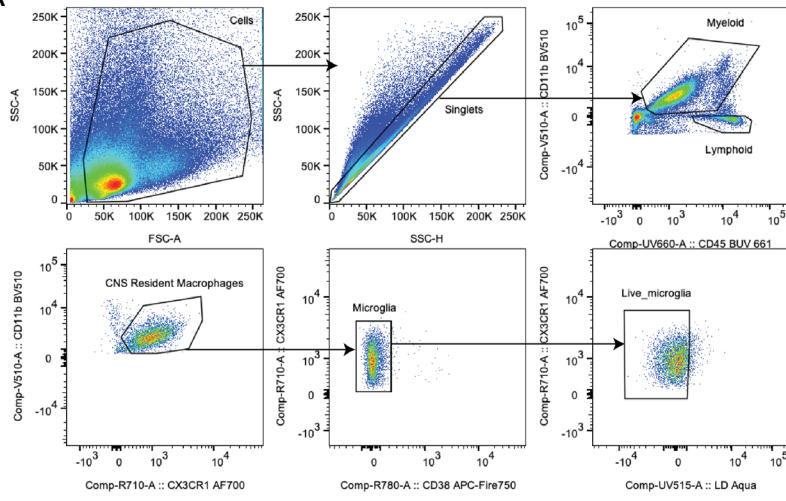**B**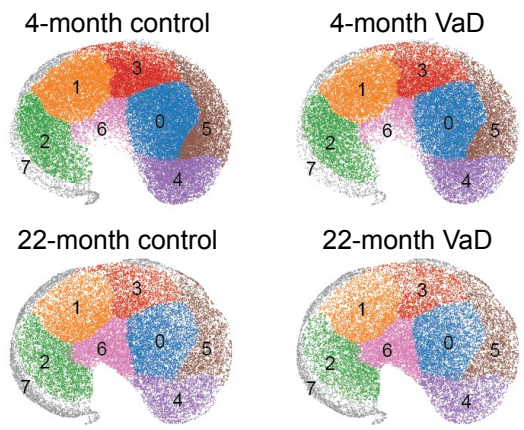**C**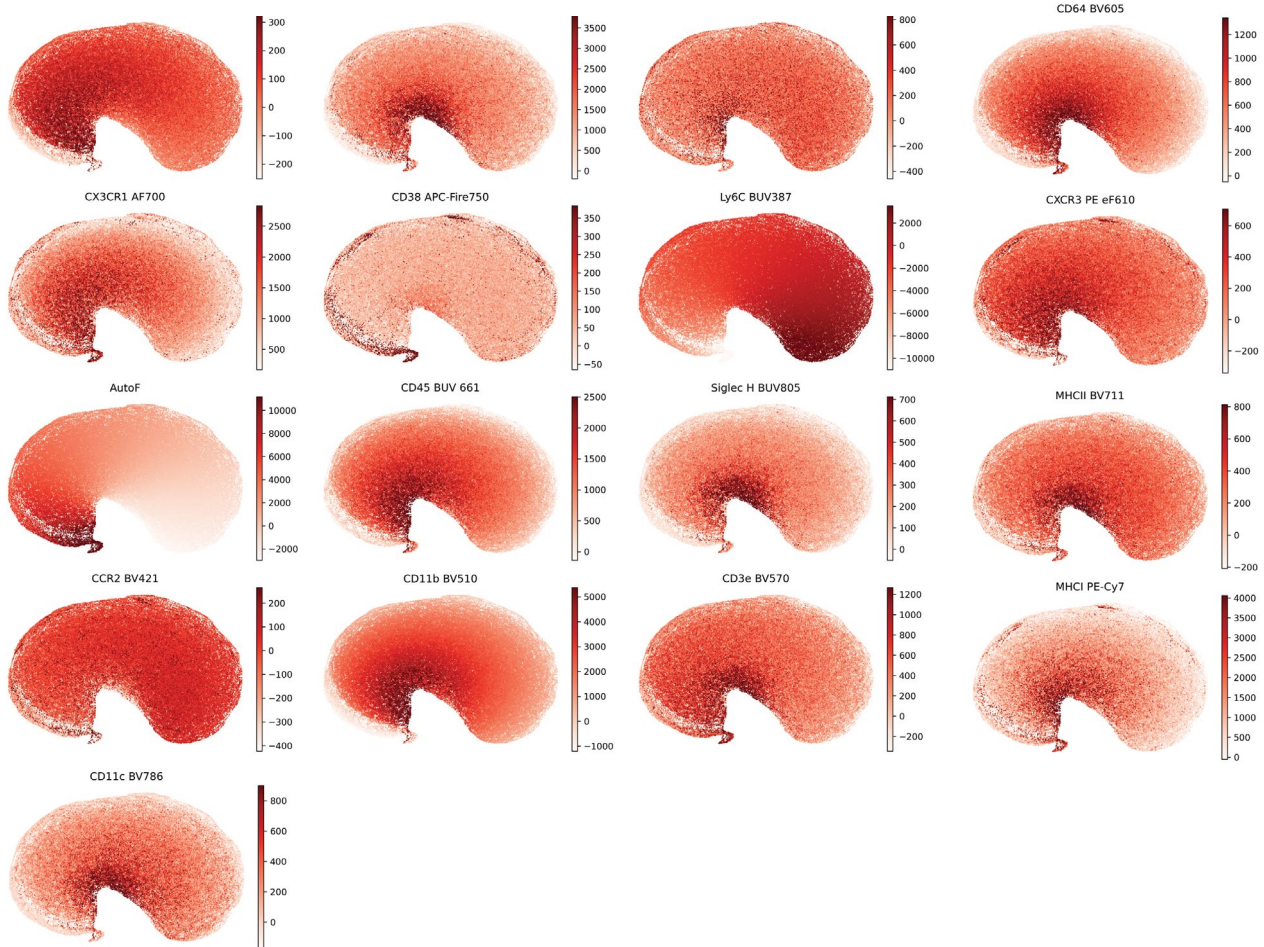**D**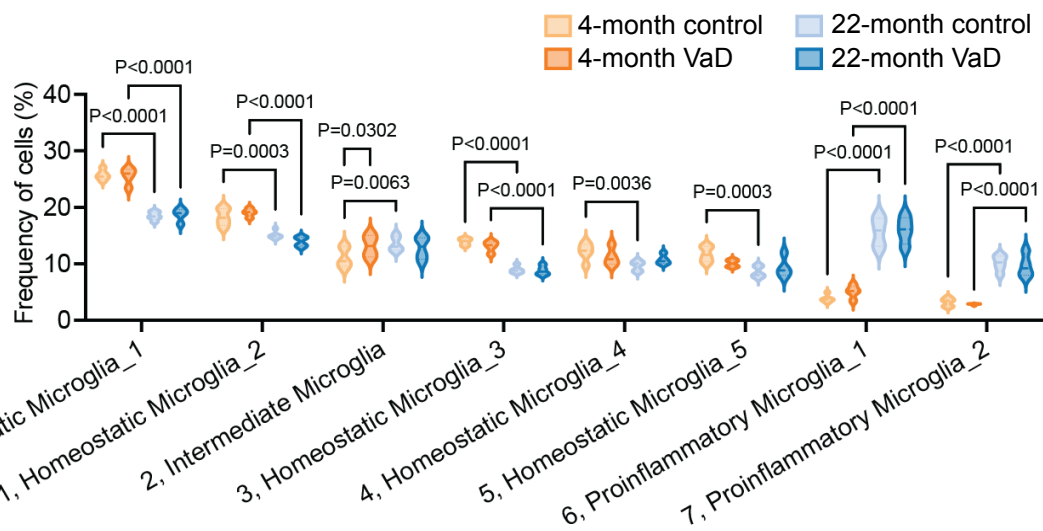

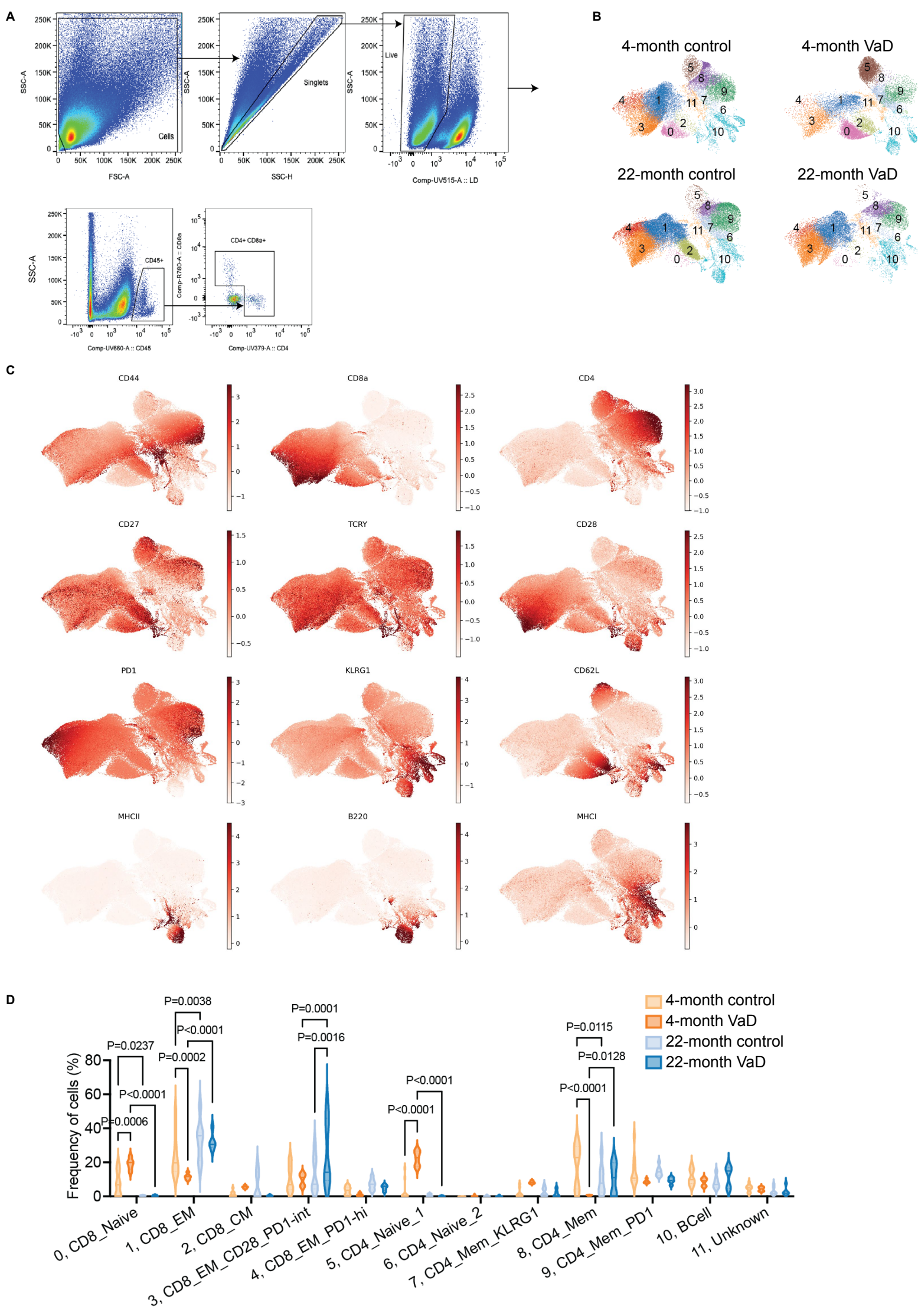

A

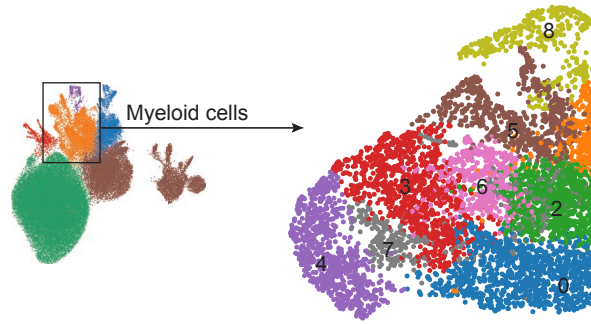

B

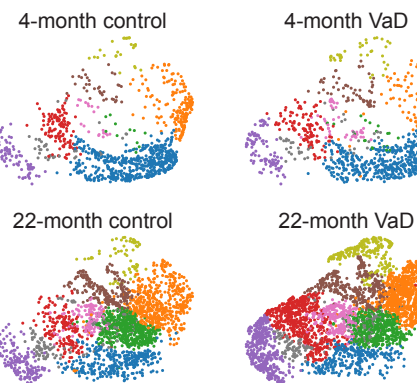

C

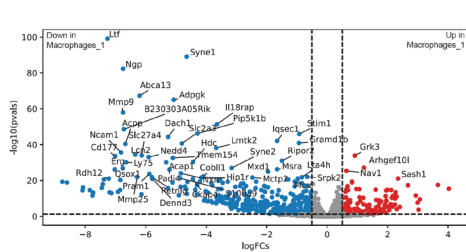

D

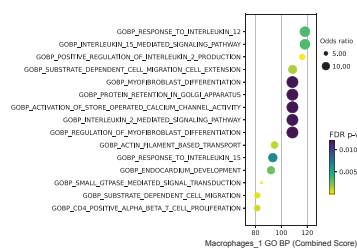

E

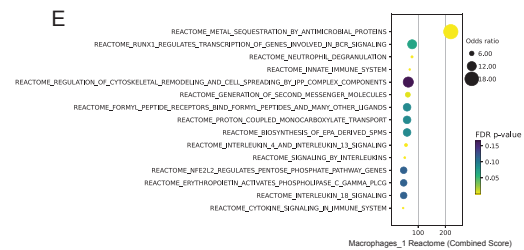

F

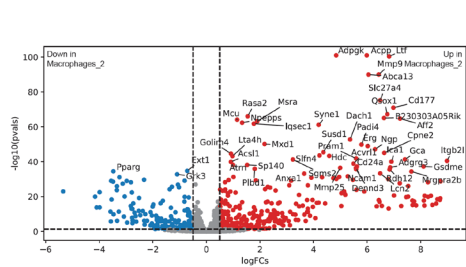

G

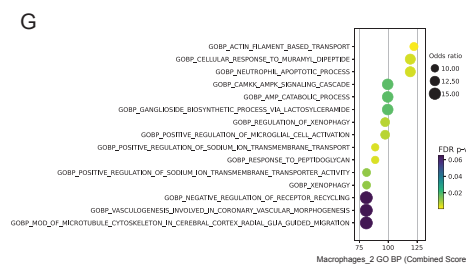

H

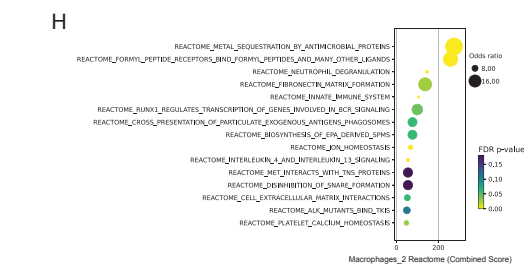

I

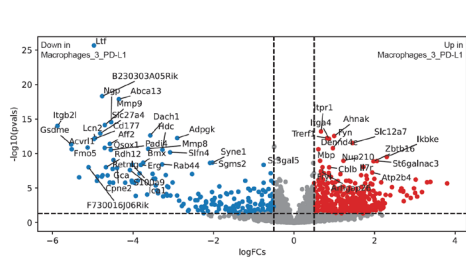

J

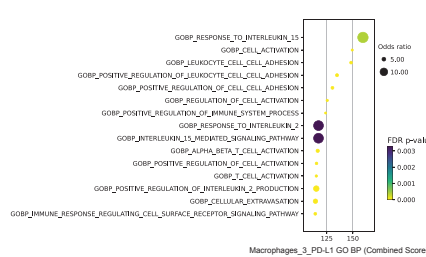

K

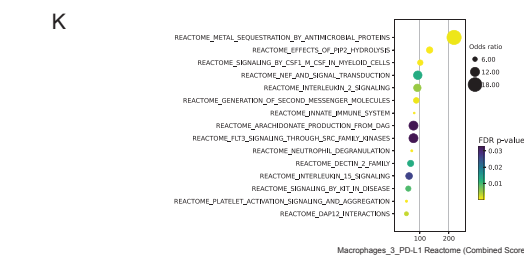

Supplement: Supplement 1 — Figure S1. Gating Strategy for Sorting Immune Cells from the Brain Flow-assisted cell sorting (FACS) of immune cells from the murine brain to isolate cells, singlets, live cells, CD45int and CD45hi resident immune cells, peripheral myeloid cells and CD3+ T cells followed by confirmation of CD8 and CD4 expression within the CD3+ T cells prior to performing barcoding for SPLiT-seq analysis. Figure S2. Spectral Flow Cytometry Corroborates Murine Brain Microglial Heterogeneity in Aging and Vascular Dementia (A) Gating strategy for identifying live singlet microglia as CD45+ CD11b+ CX3CR1+ CD38− cells. This population was then analyzed using principal components analysis (PCA), uniform manifold approximation projection (UMAP), and Leiden clustering. (B) Microglia cells shown by UMAP embedding split by age and treatment depicting Leiden cluster assignments. (C) Microglia plotted by UMAP embedding showing spectral protein counts from spectral flow cytometry data for microglial phenotypic markers. (D) Violin plots annotated by microglial cell sub-cluster classification based on protein expression quantifying eight microglial subtypes across groups. Violin plots represent mean ± quartiles. Statistical significance among groups were assessed by two-way ANOVA with Fishers Least Significant Difference post-hoc test with p-values indicated above each relevant comparison. N=4/group for VaD groups and N=5/group for control groups. n=140,296 microglia. Figure S3. Spectral Flow Cytometry Validates Murine Brain T cell Memory Accumulation with Age (B) Gating strategy for identifying live singlet T cells as CD45+ CD8a+ or CD4+ cells. This population was then analyzed using principal components analysis (PCA), uniform manifold approximation projection (UMAP), and Leiden clustering. (B) T cells shown by UMAP embedding split by age and treatment depicting Leiden cluster assignments. (C) T cells plotted by UMAP embedding showing spectral protein counts from spectral flow cytometry data for [file media-1.pdf]
